# Maternal exposure to a Western‐style diet causes differences in intestinal microbiota composition and gene expression of suckling mouse pups

**DOI:** 10.1002/mnfr.201600141

**Published:** 2016-07-12

**Authors:** Wilma T. Steegenga, Mona Mischke, Carolien Lute, Mark V. Boekschoten, Agnes Lendvai, Maurien G. M. Pruis, Henkjan J. Verkade, Bert J. M. van de Heijning, Jos Boekhorst, Harro M. Timmerman, Torsten Plösch, Michael Müller, Guido J. E. J. Hooiveld

**Affiliations:** ^1^ Nutrition, Metabolismand Genomics GroupDivision of Human NutritionWageningen UniversityWageningenThe Netherlands; ^2^ Center for LiverDigestive and Metabolic DiseasesDepartment of PediatricsUniversity Medical Center GroningenUniversity of GroningenGroningenThe Netherlands; ^3^ Nutricia ResearchUtrechtThe Netherlands; ^4^ NIZO food research BVThe Netherlands; ^5^ Department of Obstetrics and GynaecologyUniversity Medical Center GroningenUniversity of GroningenGroningenThe Netherlands; ^6^ Nutrigenomics and Systems NutritionNorwich Medical SchoolUniversity of East AngliaNorwichUK

**Keywords:** Gut development, Maternal diet, Microbiota composition, Offspring, Transcriptomics

## Abstract

**Scope:**

The long‐lasting consequences of nutritional programming during the early phase of life have become increasingly evident. The effects of maternal nutrition on the developing intestine are still underexplored.

**Methods and results:**

In this study, we observed (1) altered microbiota composition of the colonic luminal content, and (2) differential gene expression in the intestinal wall in 2‐week‐old mouse pups born from dams exposed to a Western‐style (WS) diet during the perinatal period. A sexually dimorphic effect was found for the differentially expressed genes in the offspring of WS diet‐exposed dams but no differences between male and female pups were found for the microbiota composition.

Integrative analysis of the microbiota and gene expression data revealed that the maternal WS diet independently affected gene expression and microbiota composition. However, the abundance of bacterial families not affected by the WS diet (*Bacteroidaceae, Porphyromonadaceae*, and *Lachnospiraceae*) correlated with the expression of genes playing a key role in intestinal development and functioning (e.g. *Pitx2* and *Ace2*).

**Conclusion:**

Our data reveal that maternal consumption of a WS diet during the perinatal period alters both gene expression and microbiota composition in the intestinal tract of 2‐week‐old offspring.

**Abbreviations**
CIM: clustered image map
clr: centered log ratio
DOHaD: Developmental Origin of Health and Disease
FC: fold change
IPA: ingenuity pathway analysis
IQR: interquartile range
ID: identifiers
LF: low fat
MA: microarray analysis
MO: microorganism
PCA: principal component analysis
PLS: partial least squares
PRR: pattern recognition receptor
RDA: redundancy analysis
SI: small intestine
WS: Western‐style

## 1 Introduction

The adverse health effects of maternal malnutrition during the perinatal period on the offspring are increasingly acknowledged 1. According to the Developmental Origin of Health and Disease (DOHaD) concept, perturbations of the intrauterine environment at the critical period of developmental plasticity can disrupt normal development and give rise to an altered phenotype 2. Accumulating evidence shows that maternal consumption of a Western‐style (WS) diet, rich in calories, and high in (saturated) fat and cholesterol, is an important risk factor for the development of noncommunicable diseases in the offspring during the adult phase of life. Intrauterine exposure to a WS diet has been shown to affect organogenesis of the pancreas, heart, muscle, adipose tissue, placenta, and kidney, and to alter physiological parameters such as lipid plasma profiles, inflammatory markers, and blood pressure in the offspring 3, 4, 5, 6.

The intestine is known to be of crucial importance for whole body immune/metabolic health but still is a relatively unexplored organ with respect to early‐life programming. In the small intestine (SI) food is enzymatically digested and absorbed. The largely indigestible remnants that pass the SI and enter the colon may be fermented by a community of microorganisms (MO). Following birth, colonization of the colon develops to form a complex community of bacterial species 7, 8. In humans, this community becomes firmly established during the first years of life and around the age of 3 years 9 it resembles that of adults in its composition and diversity 10, 11. Importantly, while intake of fiber‐rich plant‐derived foods affect the microbiota composition of the gut in a favorable way, the opposite has been reported following consumption of diets rich in saturated fats, added sugars or digestible starch 12, 13, 14, 15, 16, 17, 18. Exposure to a high‐saturated fat diet has been described to reduce the overall diversity and shifts the composition of the gut microbiota 19, 20, 21 resulting in “dysbiosis,” a condition indicating a microbial ecosystem where bacteria do not live in mutual accord and where potential harmful or pathogenic species predominate over those with beneficial properties. Importantly, Cox and colleagues have recently shown that altering the postnatal microbiota composition by low‐dose antibiotic exposure has permanent metabolic consequences 22. This result implies that development of an optimal microbiota composition during the early life phase is of crucial importance for metabolic health in adult life.

We and others have shown that exposure to a high‐fat diet causes alterations in gene expression in the intestine in both young adult and old mice 23, 24, 25, 26. Although the direct effects of a high‐fat diet on microbiota composition and intestinal gene expression have been extensively studied, the more indirect effects of maternal nutrition on the development of the intestine are until now largely unexplored. To overcome this gap, we have examined the SI and colon of 2‐week‐old male and female mouse pups born to mothers that were exposed to a low‐fat (LF) control diet or WS diet during the pregestation, pregnancy, and the lactation phase. Transcriptome analysis was carried out to evaluate host whole‐genome gene expression in the SI and colon of the mouse pups. The microbiota composition of the colonic luminal content of the same suckling mice was examined by 16S rRNA gene sequencing. Unlike most studies that explore the dietary effects on gene expression and microbiota composition separately, we pursued a novel approach by using recently developed multivariate statistical tools allowing integrative analysis 27 of the two sets of data.

## 2 Materials and methods

### 2.1 Animals and diets

The national and institutional guidelines for the care and use of animals were followed, and the experimental procedures were reviewed and approved by the Ethics Committee for Animal Experiments of the University of Groningen, The Netherlands (ethics registration code 5709).

**Figure 1:**
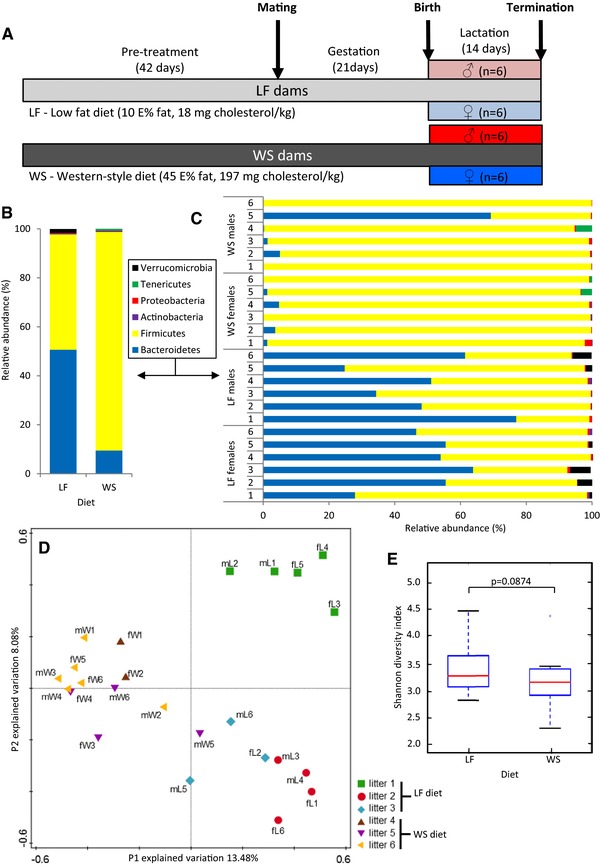
The microbiota composition of the colonic luminal content differs between the offspring of dams that were exposed to a LF or WS diet during the perinatal period. (A) Study design: C57BL/6 dams received either a low‐fat control diet (LF) or a Western‐style high fat diet (WS) throughout the study. Male and female 2‐week‐old offspring were sacrificed from LF‐fed (three independent litters) and WS diet‐exposed (three independent litters) dams. (B) Deep sequencing showed a marked decrease in the mean relative abundance of *Bacteroides* in the offspring of WS diet‐exposed dams. (C) Substantial interindividual variation was observed in the relative abundance of the six phyla measured in each individual mouse. (D) The RDA plot demonstrates clustering of the samples based on maternal diet (PC‐1) and litter in which the mouse pups were habited until scarified (PC‐2). (E) Offspring of WS diet‐exposed dams showed a nonsignificant but trend‐wise decrease in α‐diversity.

Female C57BL/6 mice (5 weeks of age) were purchased from Harlan (Horst, The Netherlands) and housed individually in the light‐ and temperature‐controlled facility of the University Medical Center Groningen (lights on 7:00 am–7:00 pm, 21°C). The mice had free access to drinking water and were randomly assigned to either a semisynthetic low‐fat control diet (LF diet, 3.85 kcal/g; 10 E% fat, 20 E% protein, 70 E% carbohydrate; D12450B, Research Diets, New Brunswick, USA) that contained low amounts of cholesterol from lard (18.0 mg cholesterol/kg), or a semisynthetic energy rich Western‐style high fat diet (WS diet, 4.73 kcal/g; 45 E% fat, 20 E% protein, 35 E% carbohydrate; D12451, Research Diets, New Brunswick, USA) that contained a high cholesterol content from lard (196.5 mg cholesterol/kg). After 6 weeks on their respective diets (pretreatment period), the female mice were mated with males that were fed the control diet. In case conceiving failed, mice were allowed to remate. Mice were allowed to deliver spontaneously and were left undisturbed with their litters for 24 h. Litter sizes were standardized to 5–7 pups to ensure no litter was nutritionally biased. The litter size of some dams was reduced further due to natural circumstances. The 2‐week‐old offspring analyzed in this study came from three litters of LF diet‐exposed dams and three litters of WS diet‐exposed dams. Equal numbers of males and females from both the LF and WS diet litters were included whereby a random selection was made to exclude 3 of the total number of 27 2‐week‐old pups. Throughout pregnancy and lactation, the dams received the same diets as during pretreatment. After 2 weeks of lactation, the offspring were sacrificed and the colon and SI were isolated from each mouse, snap‐frozen in liquid nitrogen, and stored at –80°C until further use. A schematic representation of the study design is presented in Fig. 1A. Physiological and molecular effects observed in the liver of these mice 28 and sexually dimorphic characteristics in the SI and colon under low‐fat conditions 29 have been reported previously.

### 2.2 RNA Isolation

Total RNA was isolated from the whole SI and colon (after removal of the luminal content) as described previously 29. Samples were considered suitable for hybridization when they showed intact bands of 18S and 28S ribosomal RNA subunits, displayed no chromosomal peaks or RNA degradation products, and had an RNA integrity number above 8.0.

### 2.3 Microarray hybridization and analysis

For each pup the transcriptome in SI and colon were analyzed as described previously 29 on Affymetrix GeneChip Mouse Gene 1.1 ST arrays according to standard Affymetrix protocols. Microarray quality control and normalization were performed using Bioconductor software packages integrated in an on‐line pipeline 30. Normalized expression estimates of probe sets were computed by the robust multiarray analysis algorithm available in the Bioconductor library *AffyPLM* using default settings 31. Probe sets were redefined according to Dai et al. 32 and assigned to unique gene identifiers (IDs) of the Entrez Gene database, resulting in 21,187 assigned Entrez IDs (custom CDF v17). Array data were submitted to the Gene Expression Omnibus and are available under accession number GSE72705. Of the 21,187 genes probed by the microarray analysis (MA), only genes with an intensity value of ≥20 on at least five arrays, represented by at least seven probes per gene on the array and an interquartile range (IQR) ≥0.1 were selected for further analysis. Not annotated (NA) and pancreas‐specific genes (http://biogps.org) were removed from the analysis in order to omit an effect of potential pancreatic contamination. SI and colon samples were analyzed separately. Before statistical analysis, gene expression values were log2 transformed. Differentially expressed probe sets were identified by using linear models (library *limma*) and an intensity‐based moderated *t*‐statistic 33, 34. Resulting 2log fold changes (FC) and *p*‐values were used for further descriptive bioinformatic analysis of the data. The top 1000 most variable genes were selected for principal component analysis (PCA) in MultiExperiment Viewer version 4.8.4 35, 36 with an eigenvalue of 1.0 as a cutoff for identification of contributing components. Ingenuity Pathway Analysis (IPA; Ingenuity® Systems, www.ingenuity.com) was used for functional analysis.

### 2.4 Bacterial DNA extraction and library preparation for 16S rRNA pyrosequencing

DNA was extracted from the freeze‐dried luminal content of the colon as described previously 29. Universal primers were applied (forward primer, 5′‐*CCATCTCATCCCTGCGTGTCTCCGACTCAGNNNNNN* **ACTCCTACGGGAGGCAGCAG**‐3′; reverse primer 5′‐*CCTATCCCCTGTGTGCCTTGGCAGTCTCAG* **CRRCACGAGCTGACGAC**‐3′) for amplification of the V3‐V6 region of the 16S rRNA gene as described before 29. Purified PCR products were submitted for pyrosequencing of the V3‐V4 region of the 16S rRNA gene on the 454 Life Sciences GS‐FLX platform using Titanium sequencing chemistry at GATC biotech, Konstanz, Germany.

### 2.5 16S rRNA gene sequence analysis

Deep sequencing data were analyzed with a workflow based on QIIME v1.2 37 as described before 29. Alpha diversity metrics were calculated as implemented in QIIME v1.2. The Ribosomal Database Project classifier version 2.2 was performed for taxonomic classification 38. Effects of the maternal diet on microbiome composition were analyzed by using linear models in which litter was included as categorical covariate. Before determining the FC and the statistical significance of the changes in mean relative abundance between the offspring of the LF and WS diet‐exposed dams on the different taxonomic levels, microbiome composition data was first transformed using the centered log ratio (clr) implemented in the library *Aldex2* 39, 40.

### 2.6 Multivariate integration and correlation analysis

To get an unbiased insight into the interactions between changes in gene expression and microbiota composition, the datasets were combined using the linear multivariate method partial least squares (PLS) 41. This analysis ignores diet group membership. Since we did not want to make any “a priori” assumption on the relationship between the two sets of variables that were analyzed, the canonical correlation framework of PLS was used 42. As input the 16 bacterial families with a relative abundance of at least 0.1% in one of the two intervention groups was used. In the first PLS canonical correlation analysis the top‐500 most significantly differentially expressed genes due to maternal exposure to a WS diet and for the second analysis the top‐500 IQR genes were included. Before analysis, gene expression values were log2 transformed, and microbiome composition data was transformed using the clr. Correlation matrices were visualized in clustered image maps 43. Analyses were performed using the library mixOmics 44.

## 3 Results

### 3.1 Maternal exposure to a Western‐style diet alters the microbiota composition in the offspring

The microbiota composition of the colonic luminal content of 2‐week‐old male and female C57BL/6 mice was assessed by deep sequencing of the V3‐V4 region of the 16S rRNA genes. Taxa belonging to the Bacteriodetes and Firmicutes phyla dominated the colonic luminal content in the offspring of the LF and WS diet‐exposed dams (97.7 and 98.6%, respectively). Other phyla (Actinobacteria, Proteobacteria, Tenericutes, and Verrucomicrobia) were present at very low relative abundance (Fig. 1B). A strong reduction in relative abundance of Bacteroidetes was observed in the offspring of WS diet‐exposed dams causing a pronounced increase in the Firmicutes/Bacteriodetes ratio compared to offspring of LF diet‐fed dams. Exploring the phyla composition of the 24 individual mouse pups revealed substantial interindividual variation between the mice (Fig. 1C). In all pups born from the WS diet‐fed dams the relative amount of Bacteroides was low or extremely low, except for 1 male (mW5) that displayed a Firmicutes/Bacteriodetes ratio similar to the LF diet offspring. Furthermore, Verrucomicrobia were observed in several pups of the LF diet‐exposed dams while Tenericutes were detected in two pups of the WS diet group.

To compare the overall features of the microbiota composition per individual mouse in more detail a redundancy analysis (RDA) was carried out. The RDA plot presented in Fig. 1D showed that axis P‐1 separated the LF from the WS diet offspring (explained variation: 13.48%). In addition, clustering of the different litters in which the mice were habited was detected (explained variation: 8.08%). Clustering of microbial communities from littermates in the WS diet group was less pronounced compared to the LF diet group. We have previously reported that the microbiota composition between male and female offspring of the LF diet dams did not differ significantly 29. In line with those observations, also in the offspring of WS diet‐exposed dams no significant differences were found between the two sexes (Fig. 1D and PC‐3 data not shown). The Shannon index of the offspring of the LF and WS diet‐exposed mice revealed that the α‐diversity was decreased in the WS diet offspring (Fig. 1E) but this effect was not significant (Mann–Whitney *U p*‐value 0.0874).

Next, we determined the FC and the statistical significance of the changes in mean relative abundance between the offspring of the LF and WS diet‐exposed dams on the different taxonomic levels in more detail. To circumvent a litter bias as observed the RDA plot the raw data were corrected for litter. The results presented in Table 1 show that the Bacteroidetes families *Rikenellaceae, Bacteroidaceae*, and *Porphyromonadaceae* were present in the colonic luminal content of the LF diet pups. A strong (FC > 50) and significant (*p* < 0.01) reduction in the relative abundance of *Rikenellaceae Alistipes* was found in the offspring of the WS diet‐fed dams. The Firmicute families that were most abundant in the LF offspring were *Lachnospiraceae*, *Ruminococcaceae*, and *Lactobacillaceae*. In the offspring of the WS diet‐fed dams the relative abundance of the same three families increased even further but this effect was only partially significant. Interestingly, the abundance of the Firmicute genus, *Erysipelotrichaceae Incertae Sedis*, displayed an opposite effect. This genus was present in relatively low abundance (2.0%) in the offspring of LF diet‐exposed offspring and was undetectable in the colonic luminal content of WS diet offspring. The same observation was made for Verrucomicrobia *Akkermansia—*present in low abundance (1.7%)—and Actinobacteria *Micrococcaceae—*present in extreme low abundance (0.1%)—in the offspring of LF‐diet dams, with both genera further diminishing in WS diet‐fed pups. In contrast, Teneriticus *Anaeroplasma* was found at low abundance in the pups from WS diet‐fed dams but the genus was not detected in the offspring from LF diet‐fed dams.

**Table 1:** Relative abundance of the microbiota in the colonic luminal content of the offspring of LF and WS diet exposed dams

Phylum	Class	Order	Family	Genus	LF (%)	WS (%)	FC	*p* value^a^
Bacteroidetes					49.6	6.0	–8.3	5.44 × 10^–5^
	Bacteroidetes				49.4	5.5	–9.1	3.15 × 10^–5^
		Bacteroidales			49.4	5.5	–9.1	1.17 × 10^–4^
			Rikenellaceae		13.1	0.0	–475.0	6.01 × 10^–7^
				Alistipes	13.1	0.0	–591.1	1.06 × 10^–6^
			Bacteroidaceae		24.1	1.2	–20.9	6.33 × 10^–3^
				Bacteroides	24.1	1.2	–20.9	6.33 × 10^–3^
			Porphyromonadaceae		11.5	2.9	–3.9	ns
				Parabacteroides	6.6	2.8	–2.3	ns
Firmicutes					48.0	92.7	1.9	8.16 × 10^–3^
	Clostridia				22.1	53.6	2.4	ns
		Clostridiales			22.1	53.6	2.4	ns
			Lachnospiraceae		16.3	42.9	2.6	ns
				Lachnospiraceae Incertae Sedis	2.1	3.7	1.7	ns
				Roseburia	0.7	2.2	3.0	ns
			Ruminococcaceae		5.7	10.4	1.8	ns
				Anaerotruncus	1.8	1.2	–1.6	ns
				Ruminococcaceae Incertae Sedis	0.2	1.7	9.5	3.15 × 10^–3^
	Bacilli				22.8	39.0	1.7	3.59 × 10^–8^
		Lactobacillales			22.8	39.0	1.7	1.95 × 10^–10^
			Streptococcaceae		0.3	0.3	–1.2	ns
				Streptococcus	0.3	0.2	–1.3	ns
			Lactobacillaceae		22.2	38.3	1.7	8.06 × 10^–7^
				Lactobacillus	22.0	37.9	1.7	2.04 × 10^–6^
			Enterococcaceae		0.2	0.3	1.2	ns
				Enterococcus	0.2	0.3	1.2	ns
	Erysipelotrichi				2.1	0.0	–54.2	6.67 × 10^–5^
		Erysipelotrichales			2.1	0.0	–54.2	6.39 × 10^–7^
			Erysipelotrichaceae		2.1	0.0	–54.2	3.73 × 10^–4^
				Erysipelotrichaceae Incertae Sedis	2.0	0.0	<–1500	6.75 × 10^–6^
Actinobacteria					0.4	0.4	1.0	4.80 × 10^–1^
	Actinobacteria				0.4	0.4	1.0	ns
		Actinomycetales			0.3	0.3	1.2	ns
			Micrococcineae		0.1	0.0	–90.0	3.03 × 10^–7^
				Micrococcaceae	0.1	0.0	–90.0	3.49 × 10^–7^
			Corynebacterineae		0.2	0.3	1.9	ns
				Corynebacteriaceae	0.2	0.3	1.9	ns
		Coriobacteriales			0.1	0.0	–3.2	ns
			Coriobacterineae		0.1	0.0	–3.2	ns
				Coriobacteriaceae	0.1	0.0	–3.2	ns
Proteobacteria					0.2	0.1	–1.6	2.98 × 10^–3^
	Gammaproteobacteria				0.2	0.1	–1.5	2.63 × 10^–3^
		Pasteurellales			0.0	0.1	5.4	5.09 × 10^–3^
			Pasteurellaceae		0.0	0.1	5.4	2.90 × 10^–3^
		Enterobacteriales			0.2	0.0	<–1500	ns
			Enterobacteriaceae		0.2	0.0	<–1500	ns
				Shigella	0.2	0.0	<–1500	ns
Tenericutes					0.0	0.8	279.7	1.94 × 10^–3^
	Mollicutes				0.0	0.8	279.7	3.95 × 10^–4^
		Anaeroplasmatales			0.0	0.8	279.2	3.02 × 10^–3^
			Anaeroplasmataceae		0.0	0.8	279.2	1.51 × 10^–3^
				Anaeroplasma	0.0	0.8	279.2	1.27 × 10^–3^
Verrucomicrobia					1.7	0.0	–1207.7	1.35 × 10^–3^
	Verrucomicrobiae				1.7	0.0	–1207.7	1.13 × 10^–3^
		Verrucomicrobiales			1.7	0.0	–1207.7	1.03 × 10^–3^
			Verrucomicrobiaceae		1.7	0.0	–1207.7	3.09 × 10^–3^
				Akkermansia	1.7	0.0	–1207.7	1.07 × 10^–3^

Abundance treshold for presentation in the table >0.1% in at least one of the two diet groups.

a Moderate *t*‐test.

In summary, the obtained results reveal strong differences in the microbiota composition present in the colonic luminal content of suckling offspring from LF and WS diet‐exposed dams. In addition, litter was found to strongly affect the microbiota composition of the 2‐week‐old mouse pups, whereas sex of the mice showed no effect at this age.

### 3.2 Gene expression in the small intestine and colon of suckling mice is significantly altered by a maternal Western‐style diet

**Figure 2:**
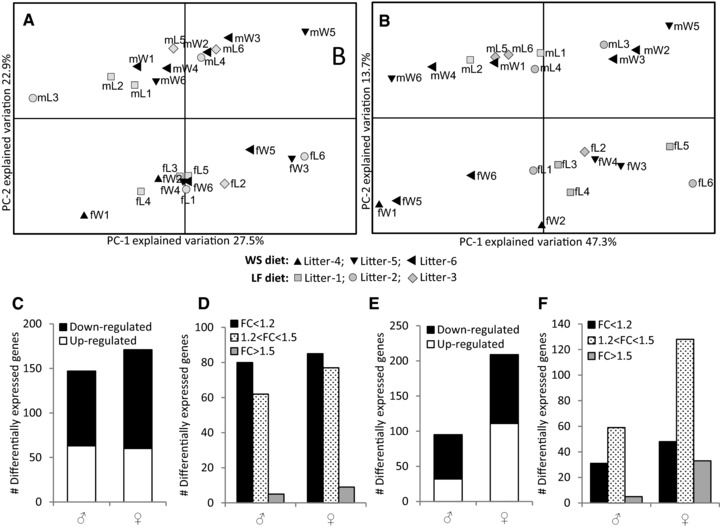
Changes in gene expression in the SI and colon of 2‐week‐old male and female C57BL/6 mice due to perinatal exposure to a WS diet. PCA of the top‐1000 most variable genes present in the (A) SI or (B) colon separates the males from females largely by PC2. (C) Significant (*p* < 0.01) differential expression in the SI in males and females. (D) The effect strength in gene expression between offspring of LF and WS diet‐exposed dams is relatively subtle in both males and females. (E) Significant (*p* < 0.01) differential expression in the colon in males and females. (F) Perinatal exposure to a WS diet caused stronger effects in the colon than in the SI in both male and female mouse pups.

To determine the effects of a perinatal maternal WS diet on gene expression in the intestine, MA was carried out on RNAs isolated from the SI and colon of the same 2‐week‐old mice. PCA of the SI (Fig. 2A) and of the colon (Fig. 2B) revealed a clear separation between males and females in both segments of the intestine. However, for both the SI and colon, PC‐1, PC‐2, and PC‐3 did not cluster the samples on the perinatal diet of the mother nor on the litters in which the mice were habited prior to sacrifice (Fig. 2A and B and for PC‐3 data not shown).

Since the results presented in Fig. 2A and B indicate that the sex of the mouse pup strongly affects gene expression, the changes in gene expression between offspring of LF and WS diet‐exposed dams were calculated independently for males and females. In the SI, 147 genes were significantly (*p* < 0.005) regulated in males and 171 in females (Fig. 2C and Supporting Information Tables S1 + S2). In both sexes the effects were subtle; the FC difference in expression between the offspring of LF or WS diet‐exposed dams was lower than 1.2 for most differentially expressed genes and only a very limited number of genes displayed a FC > 1.5 (Fig. 2D).

In the colon, 95 genes were significantly (*p* < 0.005) differentially expressed in males whereas in females 209 displayed altered expression between the offspring of LF and WS diet‐exposed dams (Fig. 2E and Supporting Information Tables S3 + S4). In both sexes, the changes induced by the perinatal maternal diet were more pronounced in the colon than in the SI (Fig. 2F).

Taken together, a maternal WS diet significantly altered gene expression in both the SI and colon of the suckling offspring. In addition, gene expression in both segments of the intestine differed strongly between both sexes.

### 3.3 Male and female offspring respond differently to a maternal WS diet

**Figure 3:**
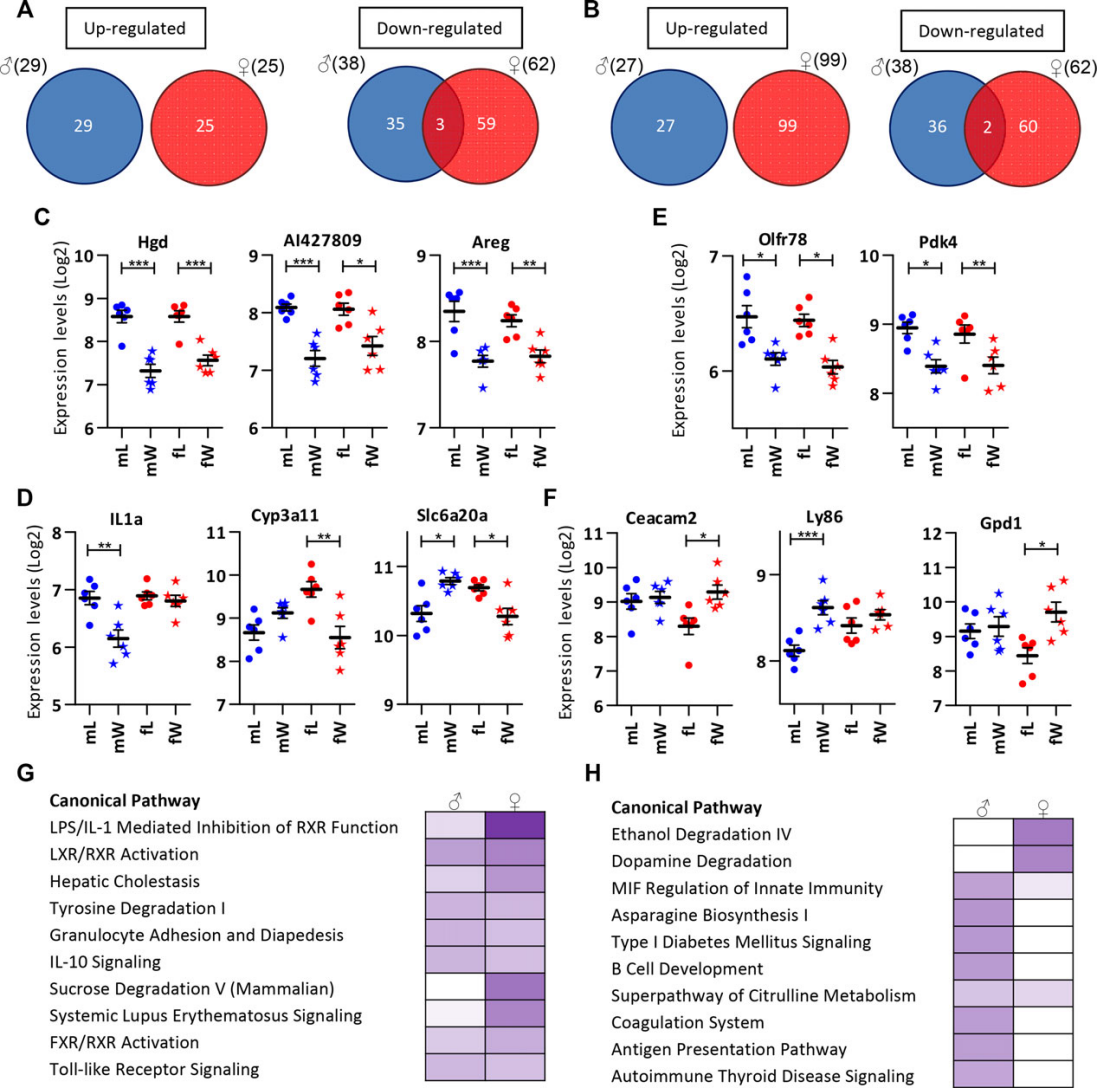
Sexually dimorphic gene expression in the SI and colon of 2‐week‐old C57BL/6 mice. A very limited overlap in differentially regulated genes due to perinatal exposure to a WS diet was found between males and females in the (A) SI and (B) colon. Expression levels of genes showing a (C) similar or (D) different WS diet‐response between males and females in the SI. Expression levels of genes showing a (E) similar or (F) different WS diet‐response between males and females in the SI. Ingenuity pathway analysis reveals similarities in the top‐10 most significantly regulated canonical pathways in males and females in the SI (G) but more distinct effects between the sexes in the colon (H). ^*^ *p* < 0.005, ^**^ *p* < 0.0005, ^***^ *p* < 0.00005.

By exploring the significant (*p* < 0.005) differences with a FC over 1.2 in response to the maternal WS diet between males and females in more detail, we found that the number of genes similarly regulated in both sexes was extremely limited in both the SI (Fig. 3A) and in the colon (Fig. 3B). In the SI, no genes were detected that were significantly upregulated in both sexes. Three genes were significantly downregulated in both males and females: *Hgd, AI427809*, and *Areg* (Fig. 3C). Examples of genes displaying a strong sexually dimorphic response were *IL1a*, *Cyp3A11*, and *Slc6a20a* (Fig. 3D). For a large subset of sexually dimorphic genes, however, the differences between the sexes appeared to be more subtle or at the borderline of significance (see Supporting Information Tables S1 + S2). In the colon no genes were upregulated in both males and females while two genes were downregulated in both sexes: *Olfr78 and Pdk4* (Fig. 3E). Examples of genes displaying a strong sexually dimorphic response are *Ceacam2*, *Ly86*, and *Gpd1* (Fig. 3F) but, similar to the SI, also in the colon many genes show subtle differences between the two sexes (Supporting Information Tables S3 + S4).

IPA was carried out to obtain insight into the functional consequences of the changes induced by a maternal WS diet for the male and female pups. The top‐10 most significantly affected canonical pathways in the SI (Fig. 3G) and colon (Fig. 3H) predominantly described metabolic processes and immune response/inflammation‐related functions. Furthermore, Fig. 3G revealed that in the SI most of the regulated pathways were affected in both sexes but to a different extent. LXR, FXR, and RXR nuclear receptor‐mediated processes were found to be affected by the WS diet in both sexes but more strongly in female offspring. In the colon (Fig. 3H), however, the contrast between pathways regulated in the two sexes was more pronounced: strong regulation of ethanol degradation IV and dopamine degradation was found in female mice but these pathways appeared unaffected in male offspring of WS diet‐exposed dams. In the contrary, a number of immune‐related pathways were found to be regulated in male but not or hardly in female offspring.

In summary, different subsets of genes were significantly affected by the maternal WS diet in males and females. Metabolic and immune response/inflammatory canonical pathways were altered by the maternal WS diet in both sexes but to a different extent.

### 3.4 Integrative analysis of genes displaying altered expression due to a maternal WS diet and luminal microbiota composition

**Figure 4:**
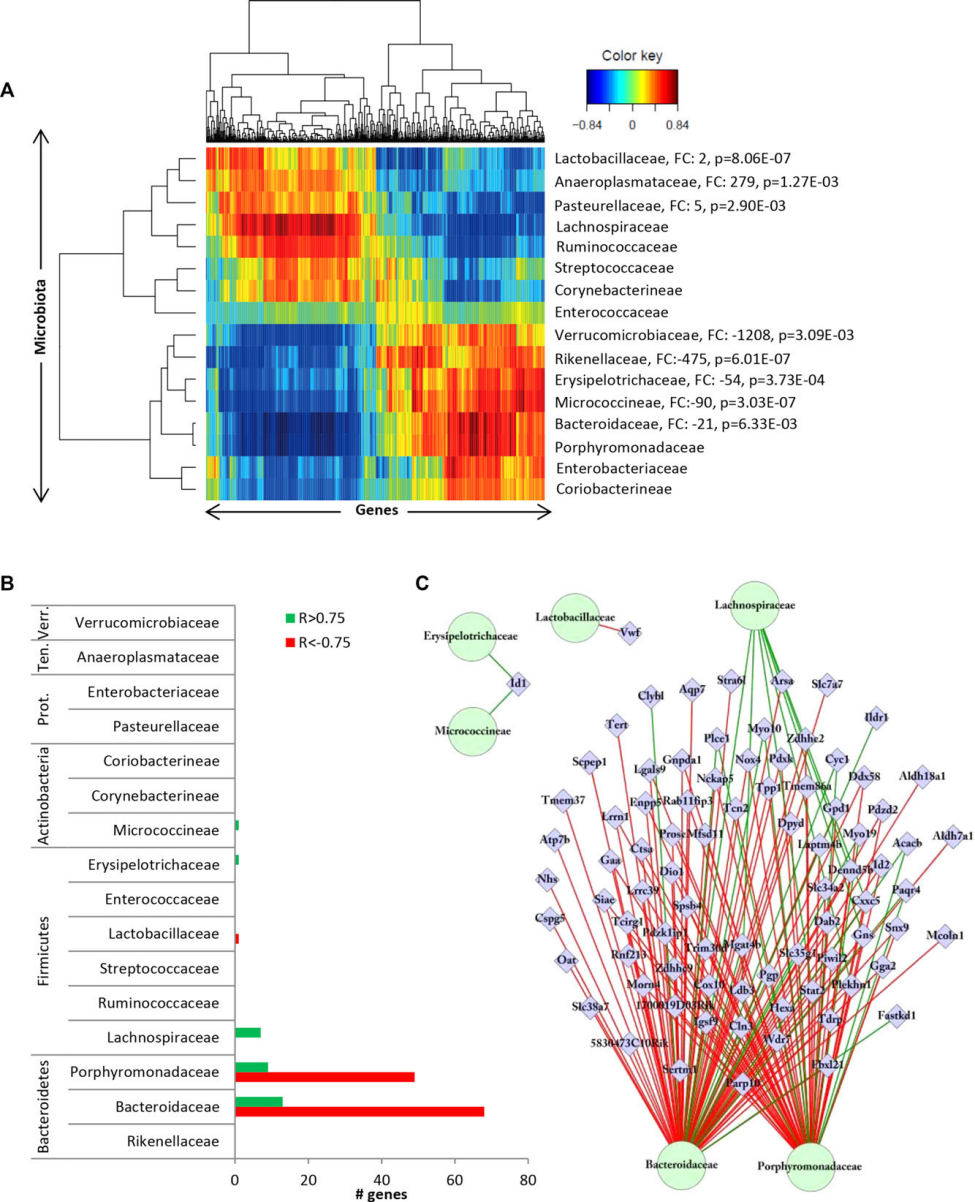
Integration of the top‐500 most significantly differentially expressed genes in the colon of males and females due to perinatal exposure by a WS diet with 16 MO families present with a the relative abundance >0.1% in the colonic luminal content of 2‐week‐old mice. The integration of datasets was performed per individual mouse and gives a direct correlation between gene expression and microbiota composition over the samples. (A) A clustered image map (CIM) demonstrates a separation of both the 16 MO families and genes into two major, similarly large clusters each, which show crosswise reverse correlation. (B) Quantification of the strong (*R* <(–0.75) or *R* > 0.75) correlation revealed that the strongest correlation was found for *Bacteroidaceae*, *Porphyromonadaceae*, and *Lachnospiraceae*. (C) Network analysis revealed a large overlap in the genes displaying a strong (*R* < −0.75 or *R* > 0.75) correlation with *Bacteroidaceae*, *Porphyromonadaceae*, and *Lachnospiraceae* and that other genes display a strong correlation with *Lactobacillaceae*, *Micrococcineae*, and *Erysipelotrichaceae*.

To reveal to what extent expression levels of genes affected by a maternal WS diet in the colonic tissue correlated with microbiota abundance in the colonic luminal content, the MA and microbiota datasets were integrated by PLS‐canonical correlation analysis. For this correlation analysis, we included the 16 microbiota families that were detected by deep sequencing with a relative abundance >0.1% in at least one of the two diet groups (listed in Table 1) and the top‐500 most significantly regulated in the colon in male and/or female mice, due to maternal exposure to a WS diet. The clustered image map (CIM) presented in Fig. 4A, representing clustering of the correlation coefficients, showed a separation of the 16 MO families into two major clusters. Correlation coefficients in the CIM were depicted with different colors and revealed that, within the two clusters, the correlation between relative microbial abundance and gene expression is rather variable. Surprisingly, the MO families displaying a strong (FC > 50) and significant (*p* < 0.01) difference in relative abundance between the offspring of LF and WS diet‐exposed dams (*Rikenellaceae*, *Erysipelotrichaceae*, *Micrococcineae*, *Anaeroplasmataceae*, and *Verrucomicrobiaceae*), did not have the highest correlation with gene expression (indicated by the most intense colors). Quantification of the strongest correlations (*R* < –0.75 and/or *R* > 0.75) between relative abundance of the bacterial families and gene expression revealed no correlations above the threshold for most of the 16 MO families (Fig. 4B and Supporting Information Table 5). On the other hand, expression of a large number of genes correlated with the Bacteriodetes families *Bacteroidaceae* and *Porphyromonadaceae* and the Firmicute family *Lachnospiraceae*. The network of these strongest correlations presented in Fig. 4C shows that all genes that correlated positively with *Lachnospiraceae* correlated negatively with *Bacteroidaceae* and/or *Porphyromonadaceae*. Interestingly, the very limited subset of genes that strongly correlated with *Lactobacillaceae*, *Erysipelotrichaceae*, and/or *Micrococcineae* did not correlate with the aforementioned Bacteroidetes and Firmicute families.

Among the top‐20 genes displaying the strongest positive and/or negative correlations (Table 2) are various candidates that might be important for intestinal functioning including genes regulating metabolic processes (i.e. *Acacb*, *Mgat4b*, *Pgp*, and *Gns*), involved in immune functioning (i.e. *Ddx58* and *Cxxc5*) or in the (structural) development of the intestine (i.e. *Id1*, *Myo10*, *Snx9*, *Dab2*, *Rab11fip3*, *Tpp1*, and *Lgals9*). Furthermore, several genes included in this list have previously been linked to colorectal cancer (i.e. *Id1*, *Lgals9*, *Stat2*, and *Dab2*).

**Table 2:** Strongest (inverse) correlations between relative abundance of microbiota and expression of genes, differentially expressed by a maternal WS diet, in the colon of 2‐week‐old mouse pups


Top R>0.75	Top R<−0.75	Symbol	Rikenellaceae	Bacteroidaceae	Porphyromonadaceae	Lachnospiraceae	Ruminococcaceae	Streptococcaceae	Lactobacillaceae	Enterococcaceae	Erysipelotrichaceae	Micrococcineae	Corynebacterineae	Coriobacterineae	Pasteurellaceae	Enterobacteriaceae	Anaeroplasmataceae	Verrucomicrobiaceae
X		Myo19	0.340	0.797	0.766	−0.721	−0.638	−0.542	−0.152	−0.071	0.261	0.353	−0.462	0.428	−0.233	0.413	−0.343	0.590
X		Id1	0.459	0.383	0.413	−0.381	−0.275	0.160	−0.425	0.131	0.767	0.790	−0.258	0.384	−0.683	0.523	−0.190	0.140
X		Cox10	0.394	0.784	0.765	−0.716	−0.632	−0.416	−0.220	−0.038	0.467	0.542	−0.502	0.496	−0.419	0.552	−0.316	0.517
X		Slc35g1	0.364	0.784	0.761	−0.712	−0.638	−0.452	−0.183	−0.055	0.418	0.493	−0.508	0.486	−0.375	0.536	−0.307	0.525
X		Paqr4	0.415	0.783	0.762	−0.717	−0.611	−0.437	−0.247	−0.023	0.380	0.472	−0.437	0.444	−0.338	0.443	−0.361	0.562
X		Cyc1	0.362	0.774	0.754	−0.703	−0.638	−0.418	−0.183	−0.054	0.482	0.548	−0.533	0.511	−0.434	0.595	−0.283	0.490
X		Myo10	0.495	0.774	0.764	−0.719	−0.585	−0.340	−0.346	0.025	0.488	0.582	−0.406	0.456	−0.432	0.459	−0.388	0.545
X		Mgat4b	0.346	0.773	0.748	−0.701	−0.627	−0.473	−0.166	−0.061	0.363	0.440	−0.488	0.460	−0.325	0.492	−0.310	0.533
X		Ildr1	0.422	0.766	0.748	−0.704	−0.595	−0.408	−0.261	−0.014	0.401	0.490	−0.427	0.442	−0.356	0.445	−0.357	0.545
X		Acacb	0.550	0.765	0.756	−0.720	−0.539	−0.346	−0.419	0.063	0.373	0.494	−0.288	0.372	−0.323	0.279	−0.469	0.613
X		Pgp	0.527	0.761	0.756	−0.713	−0.568	−0.289	−0.389	0.048	0.539	0.633	−0.391	0.459	−0.477	0.468	−0.394	0.529
X		Plce1	0.616	0.758	0.756	−0.721	−0.516	−0.274	−0.500	0.103	0.444	0.570	−0.254	0.373	−0.385	0.277	−0.496	0.606
X		Clybl	0.373	0.757	0.733	−0.691	−0.595	−0.461	−0.205	−0.039	0.313	0.402	−0.423	0.416	−0.278	0.403	−0.345	0.555
X	X	Trim30d	−0.560	−0.827	−0.811	0.774	0.585	0.427	0.411	−0.049	−0.318	−0.453	0.302	−0.376	0.274	−0.254	0.507	−0.684
X	X	Dennd5b	−0.540	−0.814	−0.800	0.760	0.591	0.397	0.390	−0.040	−0.390	−0.510	0.347	−0.412	0.340	−0.339	0.465	−0.635
X	X	Gns	−0.475	−0.823	−0.802	0.760	0.619	0.461	0.308	0.001	−0.344	−0.457	0.395	−0.424	0.302	−0.369	0.429	−0.632
X	X	Plekhn1	−0.542	−0.812	−0.799	0.758	0.591	0.385	0.392	−0.041	−0.412	−0.529	0.354	−0.421	0.360	−0.358	0.458	−0.625
X	X	Stat2	−0.454	−0.823	−0.804	0.757	0.638	0.443	0.281	0.015	−0.417	−0.515	0.452	−0.468	0.370	−0.464	0.388	−0.592
X	X	Ldb3	−0.418	−0.827	−0.803	0.756	0.651	0.482	0.236	0.037	−0.381	−0.476	0.472	−0.469	0.339	−0.469	0.372	−0.594
X	X	Cxxc5	−0.390	−0.826	−0.799	0.751	0.657	0.509	0.202	0.053	−0.350	−0.443	0.483	−0.465	0.313	−0.468	0.359	−0.594
	X	Snx9	−0.489	−0.805	−0.789	0.746	0.605	0.410	0.330	−0.013	−0.405	−0.511	0.397	−0.436	0.357	−0.403	0.417	−0.600
	X	Rab11fip3	−0.422	−0.815	−0.791	0.745	0.634	0.478	0.245	0.030	−0.353	−0.451	0.443	−0.446	0.313	−0.428	0.382	−0.599
	X	Zdhhc2	−0.463	−0.808	−0.786	0.745	0.606	0.466	0.298	0.002	−0.309	−0.423	0.376	−0.404	0.271	−0.337	0.428	−0.631
	X	Gga2	−0.371	−0.820	−0.791	0.744	0.654	0.526	0.182	0.061	−0.316	−0.409	0.479	−0.454	0.282	−0.449	0.354	−0.596
	X	Nckap5	−0.447	−0.807	−0.786	0.742	0.617	0.456	0.278	0.012	−0.352	−0.456	0.413	−0.431	0.311	−0.396	0.401	−0.605
	X	Lrrn1	−0.412	−0.812	−0.788	0.742	0.636	0.479	0.234	0.035	−0.357	−0.453	0.453	−0.451	0.317	−0.442	0.372	−0.591
	X	Mfsd11	−0.459	−0.805	−0.785	0.742	0.613	0.437	0.294	0.005	−0.377	−0.480	0.411	−0.436	0.333	−0.406	0.402	−0.598
	X	Dab2	−0.382	−0.814	−0.787	0.740	0.648	0.505	0.197	0.053	−0.339	−0.431	0.475	−0.457	0.302	−0.457	0.354	−0.587
	X	Pdzd2	−0.315	−0.823	−0.789	0.740	0.676	0.571	0.113	0.095	−0.291	−0.375	0.523	−0.468	0.262	−0.486	0.319	−0.585
	X	Lgals9	−0.415	−0.806	−0.781	0.738	0.623	0.490	0.240	0.030	−0.310	−0.412	0.422	−0.424	0.273	−0.387	0.389	−0.609
	X	Tpp1	−0.408	−0.807	−0.783	0.737	0.632	0.478	0.231	0.035	−0.352	−0.447	0.450	−0.447	0.312	−0.437	0.370	−0.588
	X	Ddx58	−0.394	−0.808	−0.780	0.737	0.630	0.513	0.214	0.043	−0.286	−0.388	0.432	−0.423	0.253	−0.388	0.380	−0.611
	X	Wdr7	−0.383	−0.804	−0.781	0.731	0.648	0.464	0.200	0.050	−0.415	−0.496	0.504	−0.487	0.372	−0.525	0.328	−0.548

*R* > 0.75.

*R* <−0.75.

In conclusion, multivariate correlation analysis revealed a strong correlation between the relative abundance of a subset of bacterial families and the expression levels of a selection of genes that are differentially expressed by a maternal WS diet.

### 3.5 Integrative analysis of genes displaying highly variable expression profiles in 2‐week‐old mice and luminal microbiota composition

**Figure 5:**
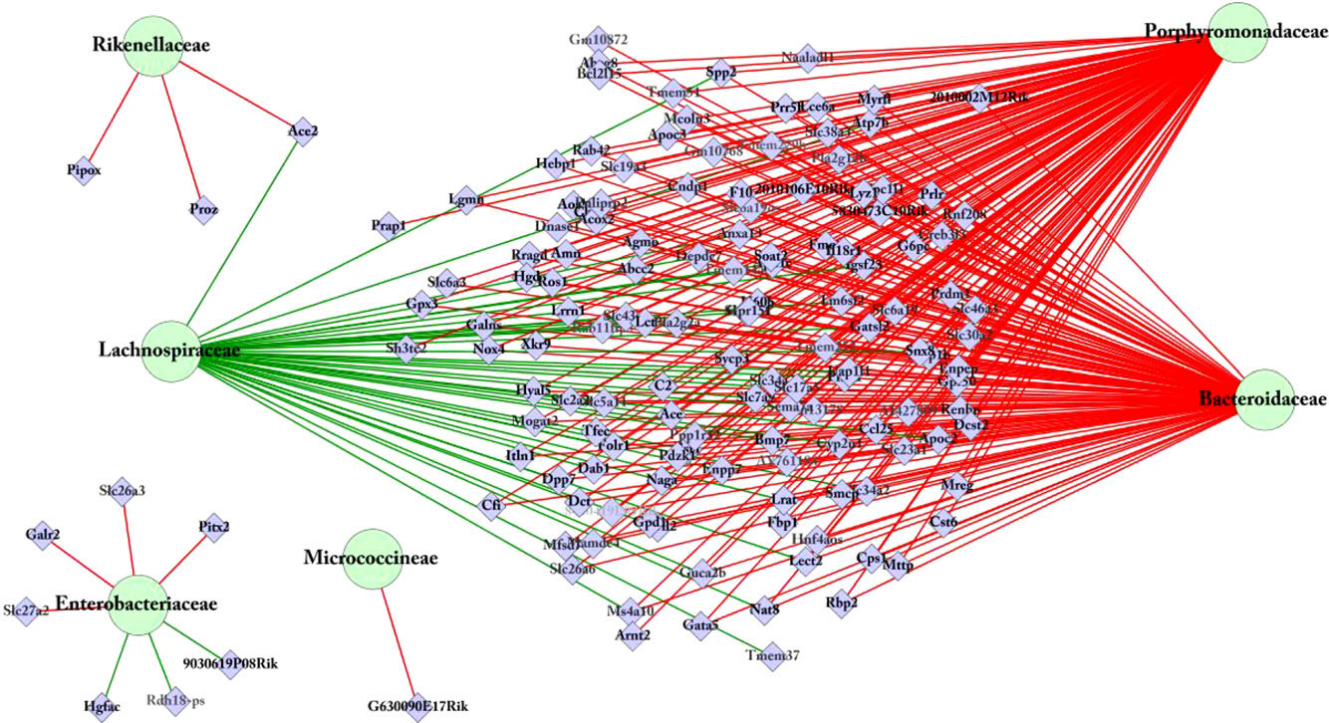
Integration of the 500 most variable genes (IQR) with 16 MO families present with a relative abundance >0.1% in the colonic luminal content of 2‐week‐old mice. Network analysis revealed a large overlap in the genes displaying a strong (*R* <(–0.75) or *R* > 0.75) correlation with *Bacteroidaceae*, *Porphyromonadaceae*, and *Lachnospiraceae* and that other genes display a strong correlation with *Rikenellaceae*, *Micrococcineae*, and *Enterobacteriaceae*.

As indicated above, PCA of the most variable genes in the colon clusters the samples on sex but did not separate the samples on the maternal diet (Fig. 2B). Comparison of the top‐500 most variable genes (IQR‐based) with the top‐500 WS diet response genes demonstrated a very limited overlap (43 genes) between the two gene sets. Since it is known that during the early postnatal phase the intestine undergoes significant structural and functional changes 45, other genes than the ones regulated by the WS diet might correlate to the microbiota composition. To explore this option, a second multivariate correlation analyses was carried out in which the same 16 microbiota families were included but now together with the top‐500 IQR genes. The obtained CIM revealed clustering of the same two groups of microbiota families (Supporting Information Fig. 1A) as found in the first correlation analysis. Quantification of the strongest correlations revealed that the same MO families (*Bacteroidaceae*, *Porphyromonadaceae*, and *Lachnospiraceae*) highly correlated to a large subset of genes and that the relative abundance of 10 of the MO families did not correlate with gene expression above the threshold (*R* < −0.75 and/or *R* > 0.75) (Supporting Information Fig. 1B and Supporting Information Table 6). In addition, *Rikenellaceae* and *Enterobacteriaceae*, families that did not show a high correlation with the WS diet‐response genes, did display positive and/or negative correlations with several genes (Fig. 5 and Supporting Information Table 6).

Evaluation of the top‐25 genes displaying the strongest variation in expression levels between the individual 2‐week‐old mouse pups (listed in Table 3) revealed that expression levels of sexually dimorphic genes such as *Xist*, *Eif2s3y*, *Uty*, *Ddx3y*, and *Kdm5d* 29 did not correlate strongly with the MO families. Interestingly, a large number of genes in the top‐25 most variable genes were previously linked to structural or functional properties of the intestine. Furthermore, expression of a subset of these genes highly correlates with the relative abundance of *Bacteroidaceae*, *Porphyromonadaceae*, and/or *Lachnospiraceae*. This result implies that these bacterial families might affect gene expression levels or, vice versa, the genes affect the abundance of these bacterial families.

**Table 3:** Correlation analysis of the Top‐25 genes with the highest IQR

Nr	Symbol	Description	Chr	*R* > 0.75 with^a^	*R* <−0.75 with^a^
1	Xist	Inactive X‐specific transcripts	X	—	—
2	Eif2s3y	Eukaryotic translation initiation factor 2, subunit 3, structural gene Y‐linked	Y	—	—
3	Uty	Ubiquitously transcribed tetratricopeptide repeat gene, Y chromosome	Y	—	—
4	Ddx3y	DEAD (Asp‐Glu‐Ala‐Asp) box polypeptide 3, Y‐linked	Y	—	—
5	Kdm5d	Lysine (K)‐specific demethylase 5D	Y	—	—
6	Lect2^b^	Leukocyte cell‐derived chemotaxin 2	13	L	B,P
7	Gm10768	Predicted gene 10768	19	—	B,P
8	G6pc^b^	Glucose‐6‐phosphatase, catalytic	11	—	B,P
9	Lct^b^	Lactase	1	L	B,P
10	Sval1	Seminal vesicle antigen‐like 1	6	—	–
11	Lyz1^b^	Lysozyme 1	10	—	B,P
12	Fgf15^b^	Fibroblast growth factor 15	7	—	—
13	Hyal5	Hyaluronoglucosaminidase 5	6	—	B,P
14	Saa1^b^	Serum amyloid A 1	7	—	—
15	Fabp6^b^	Fatty acid binding protein 6, ileal (gastrotropin)	11	—	—
16	Abcc2^b^	ATP‐binding cassette, subfamily C (CFTR/MRP), member 2	19	—	B,P
17	Slc6a19^b^	Solute carrier family 6 (neurotransmitter transporter), member 19	13	—	B,P
18	Mamdc4	MAM domain containing 4	2	—	B,P
19	Olfm4^b^	olfactomedin 4	14	—	—
20	Npl^b^	N‐acetylneuraminate pyruvate lyase	1	—	—
21	Mfi2^b^	Antigen p97 (melanoma associated)	16	—	—
22	Abcg5^b^	ATP‐binding cassette, sub‐family G (WHITE), member 5	17	—	—
23	Smcp	Sperm mitochondria‐associated cysteine‐rich protein	3	—	B,P
24	Slc23a1^b^	Solute carrier family 23 (nucleobase transporters), member 1	18	—	B,P
25	Lgals2	Lectin, galactose‐binding, soluble 2	15	—	—

a PLS‐based canonical correlation.

b Previously reported to be related to the intestine.

L, Lachnospiraceae; B, Bacteroidaceae; P, Porphyromonadaceae.

Taken together, strong correlations were found between different bacterial families and genes displaying high variation in expression in 2‐week‐old mouse pups of which many have previously been shown to be important for the structural and functional characteristics of the intestine.

## 4 Discussion

The impact of the maternal diet on the development of the offspring is generally accepted 46, 47, 48 but the effects of a maternal WS diet on the developing intestine were, till now, still largely unknown. The results obtained in this study clearly showed that maternal exposure to a WS diet during the perinatal period significantly alters gene expression in the SI and colon of suckling, 2‐week‐old mice. Canonical pathways representing metabolic processes, immune responses, and nuclear receptor‐regulated processes were altered in the offspring of the WS diet‐exposed dams in the SI and/or colon. The subset of significantly differentially expressed genes included genes with specific functions crucial for normal functioning of the intestine, including hormones (i.e. *Cck*), transporters (i.e. *Abca1* and *Slc2a5*), nuclear receptors (*Nr1i3* and *Gpr55*), and immune response/inflammation‐related genes (*Cxcr6*, *Ly86*, *Oasl2*, and *Cd74*). These results support the concept that indirect exposure to a WS diet during the early phase of life might seriously affect the normal functioning of both segments of the intestine and that healthy nutrition of soon‐to‐be and young mothers are important for the developing offspring. It should be noticed that, although the affected pathways partially overlap between males and females, striking sexually dimorphic effects in the gene expression profiles were observed.

Substantial colonization of the intestinal lumen in humans starts at the moment of delivery, ultimately resulting in a microflora more or less stable in composition after 2–5 years. A myriad of factors including mode of delivery, breast, or bottle milk feeding, antibiotic use, BMI of the mother and genetic factors have been demonstrated to affect the settlers that develop into a complex community of bacterial species during the postnatal period 8. The crucial importance of the gut for the immune and metabolic programming of the neonate is currently commonly appreciated. Importantly, recent evidence indicates that altering the intestinal microbiota composition in early life can have long lasting metabolic consequences 22. The microbiota composition observed in the offspring of WS diet‐fed dams displayed several features that have previously been linked to exposure to a high‐fat diet at older age including (1) a strongly increased Firmicutes/Bacteroidetes ratio, (2) a (trend wise) decreased alpha‐diversity, and (3) a robust (FC>50) significant (*p* < 0.01) induction (*Anaeroplasmataceae*) or reduction (*Rikenellaceae, Erysipelotrichaceae, Micrococcineae, Enterobacteriaceae*, and *Verrucomicrobiaceae*) of bacterial families. The strong decrease in relative abundance of the *Verrucomicrobiaceae* member *Akkermansia* in the offspring of WS diet‐fed dams is in line with previous studies in older mice after exposure to a high‐fat diet 49. *Akkermansia*, residing in the outer mucus layer of the colon, has been proposed as a marker for a healthy intestine due to its reduction in disease but high abundance in the healthy mucosa 50, 51, 52. A protective or anti‐inflammatory role in the intestinal mucosa has been proposed for this bacterium and reduced abundance of *Akkermansia* in humans has been previously shown in obese individuals and people with ulcerative colitis and Crohn's disease, underscoring the importance of this bacterium for gut health 52. Our PLS‐based canonical correlation approach, however, did reveal that the relative abundance of this bacterium did not correlate strongly with gene expression. This observation implies that *Akkermansia* might be important for gut health by interacting with mucal glycoproteins but does not impact intestinal gene expression. Importantly, also other bacterial families indicated above that display a strong and significant change in relative abundance in the offspring of WS diet‐exposed dams, failed to show a strong correlation with gene expression in our integrative analysis. On the other hand, *Bacteroidaceae*, *Porphyromonadaceae*, and *Lachnospiraceae* did display a strong correlation with genes showing altered expression in the colon following perinatal exposure to a WS diet.

It is important to realize that the selection of differentially expressed genes and bacterial families with an altered relative abundance are mean values of groups whereas for the multivariate correlation analyses, data from individual mice are used. The fact that for both the MO composition (Fig. 1C) and gene expression levels (Fig. 2E+F and Supporting Information Tables S3 + S4), a strong interindividual variation was observed might contribute to the discrepancy of the families identified by the two types of analysis. However, it might also be that microbiota families other than the ones displaying the most prominent change upon exposure to a WS diet affect gene expression.

Different mechanisms have been reported via which colonic bacteria can interact with the host and vice versa. A direct interaction can be established via the so‐called pathogen‐associated molecular patterns that are common structures present on microbial surfaces. These structures can be recognized by pattern recognition receptors (PRRs) present on the immune and/or intestinal epithelial cells of the host 52. The best characterized PRRs are the Toll‐like receptors and nucleotide oligomerization domain‐like receptors 53. These receptors, however, did not display differential expression between the offspring of LF and WS diet‐exposed mice (data not shown), making it unlikely that the observed correlation between bacterial abundance and gene expression is regulated via this mechanism. However, our integrative analysis showed a strong correlation between *Anxa13* and *Bacteroidaceae*, *Porphyromonadaceae*, and *Lachnospiraceae*. *Anaxa13* has previously been identified as a receptor‐like molecule on human intestinal epithelial cells interacting with an adhesion factor from *Lactobacillus reuteri* 54. Our result implies that this gene product might also interact with other gut bacteria but further research is required to confirm this observation. An alternative mechanism by which colonic bacteria can establish a biological response in the host is by the variety of fermentation products produced by the microbiota including short‐chain fatty acids (SCFAs), ethanol, trimethylamine, acetaldehyde, and inflammatory mediators 55. In particular SCFA produced by specific colonic anaerobic bacteria have been intensively investigated in this respect 56, but the limited amount of material isolated from the colonic luminal content of the 2‐week‐old mouse pups did not allow us to measure the SCFA concentrations in this study. It is important to realize that the results obtained by the correlation analysis do not reveal a causal relation and further studies are required to explore the mechanism underlying the correlation between gene expression and altered relative abundance of specific bacterial families.

Exposure to the maternal WS diet started 6 weeks premating and lasted to the sacrifice of the 2‐week‐old mouse pups. This study does not allow us to determine whether the effect of the maternal diet is prominent during all phases or whether the effect is predominantly caused during either the premating, pregnancy, or lactation period. The period from conception to birth is characterized by rapid growth, cellular replication, and differentiation, and functional maturation of all organ systems. During the early postnatal phase the intestine undergoes significant structural and functional changes 45. Directly after birth, these changes are strongly affected by the abrupt transition in nutrient supply changing from maternal umbilical cord blood to enteral exposure to milk 57. Intriguingly, the genes displaying strong interindividual variation between the 24 male and female mice in our study were only to a limited extent WS diet‐response genes. The strongest variable genes in our dataset were five sexually dimorphic genes (*Xist*, *Eif2s3y*, *Uty*, *Ddx3y*, and *Kdm5d*) 29. PLS‐based canonical correlation approach revealed a very low correlation between the expression of these sexually dimorphic genes and any of the 16 MO families included in the analysis. Interestingly, amongst the most variable genes were many genes that were previously found to be related to the function or structure of the intestine, including *Lect2*, *G6pc*, *Lct*, *Saa1*, *Abcc2*, *Slc6a19*, *Abcg5*, and *Slc23a1*. These genes strongly correlated with the relative abundance of *Bacteroidaceae*, *Porphyromonadaceae*, and/or *Lachnospiraceae*. Interestingly, a strong correlation was found between the relative abundance of *Enterobacteriaceae* and *Pitx2*, a transcription factor that has been identified as a key regulator of left‐right asymmetry in the developing gut 58, 59. Furthermore, the Firmicute families *Rikenellaceae* and *Lachnospiraceae* demonstrated a strong correlation with the expression of *Ace2*. *Ace2* has previously been shown to be a key factor in the maintenance of intestinal homeostasis 60 and link to intestinal microbial ecology and inflammation 61, 62.

In conclusion, our data show that in 2‐week‐old suckling mouse pups that, during their short lifetime have been completely dependent on the nutrient supply by their mother, a maternal WS diet strongly affects gene expression and microbiota composition in the intestine. With respect to the DOHaD concept, our data suggest that consumption of the popular WS diet might have profound consequences for the offspring and that healthy food choices of soon‐to‐be and young mothers are important for the appropriate development of the intestine in the offspring.

*Bert J.M. van de Heijning is affiliated with Nutricia Research at the time point of submission. Jos Boekhorst and Harro Timmerman are affiliated with NIZO food research BV. The other authors declare that they have no competing interests*.

## Supporting information

Supporting information

Supporting information

Supporting information

Supporting information

Supporting information

Supporting information

Supporting information
