# Supplementary material for: Maternal exposure to a Western‐style diet causes differences in intestinal microbiota composition and gene expression of suckling mouse pups
Source: Mol Nutr Food Res. 2016 Jul 12;61(1):1600141. doi: 10.1002/mnfr.201600141 (PMC5215441; doi:10.1002/mnfr.201600141)
Supplement: Supplementary file 1 — Supporting information [file MNFR-61-0-s001.pptx]

## Slide 1
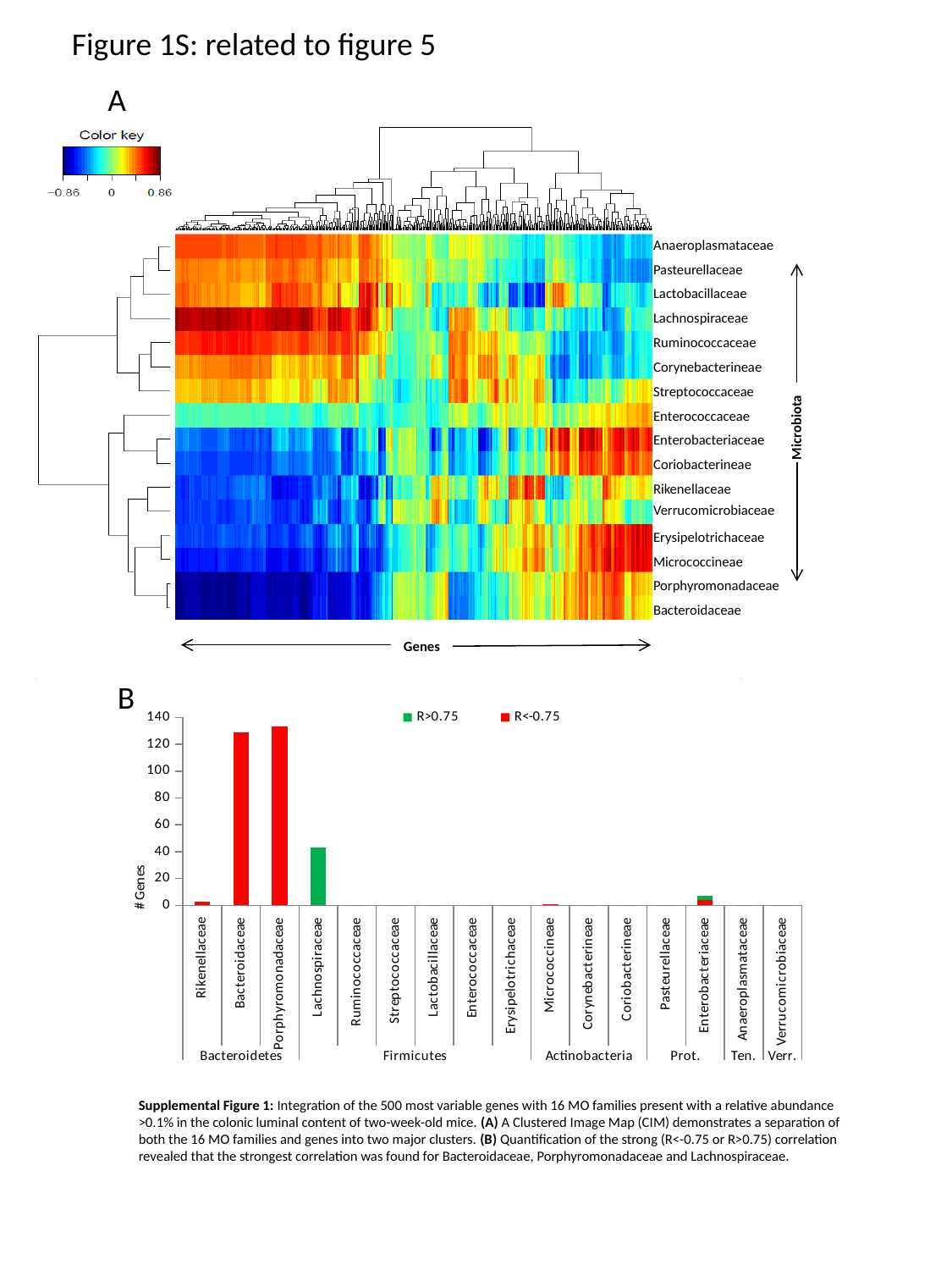

Figure 1S: related to figure 5
A
Anaeroplasmataceae
Pasteurellaceae
Lactobacillaceae
Lachnospiraceae
Ruminococcaceae
Corynebacterineae
Streptococcaceae
Enterococcaceae
Microbiota
Enterobacteriaceae
Coriobacterineae
Rikenellaceae
Verrucomicrobiaceae
Erysipelotrichaceae
Micrococcineae
Porphyromonadaceae
Bacteroidaceae
Genes
B
### Chart
| Category | R<-0.75 | R>0.75 |
|---|---|---|
| Rikenellaceae | 3.0 | 0.0 |
| Bacteroidaceae | 129.0 | 0.0 |
| Porphyromonadaceae | 133.0 | 0.0 |
| Lachnospiraceae | 0.0 | 43.0 |
| Ruminococcaceae | 0.0 | 0.0 |
| Streptococcaceae | 0.0 | 0.0 |
| Lactobacillaceae | 0.0 | 0.0 |
| Enterococcaceae | 0.0 | 0.0 |
| Erysipelotrichaceae | 0.0 | 0.0 |
| Micrococcineae | 1.0 | 0.0 |
| Corynebacterineae | 0.0 | 0.0 |
| Coriobacterineae | 0.0 | 0.0 |
| Pasteurellaceae | 0.0 | 0.0 |
| Enterobacteriaceae | 4.0 | 3.0 |
| Anaeroplasmataceae | 0.0 | 0.0 |
| Verrucomicrobiaceae | 0.0 | 0.0 |Supplemental Figure 1: Integration of the 500 most variable genes with 16 MO families present with a relative abundance >0.1% in the colonic luminal content of two-week-old mice. (A) A Clustered Image Map (CIM) demonstrates a separation of both the 16 MO families and genes into two major clusters. (B) Quantification of the strong (R<-0.75 or R>0.75) correlation revealed that the strongest correlation was found for Bacteroidaceae, Porphyromonadaceae and Lachnospiraceae.
